# Cell Origins of High-Grade Serous Ovarian Cancer

**DOI:** 10.3390/cancers10110433

**Published:** 2018-11-12

**Authors:** Jaeyeon Kim, Eun Young Park, Olga Kim, Jeanne M. Schilder, Donna M. Coffey, Chi-Heum Cho, Robert C. Bast

**Affiliations:** 1 Department of Biochemistry and Molecular Biology, Indiana University School of Medicine, Indianapolis, IN 46202, USA; ep11@iu.edu (E.Y.P.); olgakim@iu.edu (O.K.); 2 Department of Obstetrics and Gynecology, Indiana University School of Medicine, Indianapolis, IN 46202, USA; jschilde@iupui.edu; 3 Indiana University Melvin & Bren Simon Cancer Center, Indianapolis, IN 46202, USA; 4 Department of Pathology and Genomic Medicine, Houston Methodist and Weill Cornell Medical College, Houston, TX 77030, USA; dcoffey@houstonmethodist.org; 5 Department of Obstetrics and Gynecology, School of Medicine, Keimyung University, Daegu 41931, Korea; c0035@dsmc.or.kr; 6 Department of Experimental Therapeutics, The University of Texas MD Anderson Cancer Center, Houston, TX 77030, USA; rbast@mdanderson.org

**Keywords:** ovarian cancer, epithelial ovarian cancer, high-grade serous ovarian cancer (HGSOC), high-grade serous carcinoma (HGSC), ovarian cancer origin, fallopian tube, ovarian surface epithelium (OSE), serous tubal intraepithelial carcinoma (STIC)

## Abstract

High-grade serous ovarian cancer, also known as high-grade serous carcinoma (HGSC), is the most common and deadliest type of ovarian cancer. HGSC appears to arise from the ovary, fallopian tube, or peritoneum. As most HGSC cases present with widespread peritoneal metastases, it is often not clear where HGSC truly originates. Traditionally, the ovarian surface epithelium (OSE) was long believed to be the origin of HGSC. Since the late 1990s, the fallopian tube epithelium has emerged as a potential primary origin of HGSC. Particularly, serous tubal intraepithelial carcinoma (STIC), a noninvasive tumor lesion formed preferentially in the distal fallopian tube epithelium, was proposed as a precursor for HGSC. It was hypothesized that STIC lesions would progress, over time, to malignant and metastatic HGSC, arising from the fallopian tube or after implanting on the ovary or peritoneum. Many clinical studies and several mouse models support the fallopian tube STIC origin of HGSC. Current evidence indicates that STIC may serve as a precursor for HGSC in high-risk women carrying germline *BRCA1* or *2* mutations. Yet not all STIC lesions appear to progress to clinical HGSCs, nor would all HGSCs arise from STIC lesions, even in high-risk women. Moreover, the clinical importance of STIC remains less clear in women in the general population, in which 85–90% of all HGSCs arise. Recently, increasing attention has been brought to the possibility that many potential precursor or premalignant lesions, though composed of microscopically—and genetically—cancerous cells, do not advance to malignant tumors or lethal malignancies. Hence, rigorous causal evidence would be crucial to establish that STIC is a bona fide premalignant lesion for metastatic HGSC. While not all STICs may transform into malignant tumors, these lesions are clearly associated with increased risk for HGSC. Identification of the molecular characteristics of STICs that predict their malignant potential and clinical behavior would bolster the clinical importance of STIC. Also, as STIC lesions alone cannot account for all HGSCs, other potential cellular origins of HGSC need to be investigated. The fallopian tube stroma in mice, for instance, has been shown to be capable of giving rise to metastatic HGSC, which faithfully recapitulates the clinical behavior and molecular aspect of human HGSC. Elucidating the precise cell(s) of origin of HGSC will be critical for improving the early detection and prevention of ovarian cancer, ultimately reducing ovarian cancer mortality.

## 1. Ovarian Cancer

“Ovarian cancer” is an umbrella term that refers to a heterogeneous group of malignancies arising from or involving the ovary [1,2,3]. Morphologically, ovarian cancer is classified into two broad categories: (i) non-epithelial ovarian cancer (NEOC) and (ii) epithelial ovarian cancer (EOC). There are two types of NEOC: germ-cell tumors (GCT) and sex cord-stromal tumors (SCST) [4,5,6]. While 10–15% of ovarian cancer cases are NEOC [4,5], the vast majority (85–90%) belong to EOC [2,3]. According to morphology, molecular alterations, and clinical behavior, EOC is further divided into two groups: type I and type II [7]. Type I tumors are low-grade, slow-growing ovarian carcinomas. Type II tumors are high-grade, aggressive malignancies. The most common type II malignancy is high-grade serous ovarian cancer, also known as high-grade serous carcinoma (HGSC). Hence, an alternative, clinically useful way to categorize EOCs would be to simply split them into two groups: high-grade serous ovarian cancer (HGSC) and non-high-grade-serous ovarian cancer (non-HGSC). Though both are epithelial ovarian cancers, these two groups are biologically-distinct malignancies [8]. Non-HGSCs are mostly indolent tumors confined to the ovary at the time of diagnosis [9]. In contrast, HGSC is an inherently aggressive malignancy, which commonly presents as advanced-stage disease and accounts for the majority of ovarian cancer deaths [9,10,11,12,13,14]. Thus, “ovarian cancer” is also largely synonymous with “high-grade serous ovarian cancer (HGSC).” While significant tumor burden often involves the ovaries at the time of diagnosis, also typical is widespread metastatic disease involving the fallopian tubes, peritoneal surfaces, and omentum, obscuring the tissue and cell of origin of ovarian cancer [7,15,16].

### 1.1. Non-Epithelial Ovarian Cancer (NEOC)

Implicit in the name, “ovarian cancer” was thought to be tumors originating in the ovary [17,18,19]. Certainly, the ovary can be a site of tumor origin. The ovary is composed of follicles, each containing an egg, embedded in interstitial (stromal) tissue and encircled by a single layer of the ovarian surface epithelium (OSE) [20,21]. During each menstrual cycle, in response to the pituitary gonadotropins follicle-stimulating hormone (FSH) and luteinizing hormone (LH), a cohort of preantral follicles in the ovary grow to become antral follicles with multiple layers of granulosa cells and theca cells [21,22,23,24,25,26,27]. In antral follicles, the granulosa cells, theca cells, and stromal cells together make up somatic cells of the ovary [21]. These ovarian somatic cells, particularly granulosa cells (90% of SCSTs) as well as theca cells, can transform into sex cord-stromal tumors (SCST), which represent 5–8% of all primary ovarian tumors [4,28,29,30]. Ovarian tumors can also arise from the egg, which leads to germ-cell tumors (GCT) [6,31,32,33]. Germ-cell tumors and sex cord-stromal tumors are major types of non-epithelial ovarian malignancies, which account for 10–15% of all ovarian tumors [4,5]. Non-epithelial ovarian cancer (NEOC) is generally diagnosed at an early stage, where tumors are confined to the ovary without distant metastasis (GCT: 60–70%; SCST: 60–95% of cases) [4,5]. Early-stage diagnosis and chemosensitivity present a favorable prognosis to patients with NEOC (five-year survival rates: 90–100%) [4,5,28,34]. 

### 1.2. Epithelial Ovarian Cancer (EOC)

Besides non-epithelial tumors, epithelial tumors can form in the ovary. The vast majority of ovarian tumors are epithelial ovarian cancer (EOC), which accounts for 85–90% of ovarian malignancies [2,3]. According to morphologic resemblance to normal epithelial cells lining the reproductive tract, EOC can be subdivided into four major types: serous (~70%), endometrioid (10%), mucinous (3–10%), and clear-cell carcinomas (10%) [2,3,10,11,14,35]. Serous carcinoma cells resemble fallopian tube epithelium; endometrioid carcinoma is likened to endometrial epithelium; mucinous carcinoma resembles the epithelium in the endocervix; and clear-cell carcinoma is similar to clumps of normal glycogen-rich epithelial cells found in the vagina [2,3]. Also, serous carcinomas are of two types: high grade (90–96%) and low grade (4–10%) [7,14].

Recent advances in molecular and genetic analyses on ovarian carcinomas, in conjunction with clinical behavior and histopathology, have EOCs classified into type I and type II [7,36,37,38]. Type I tumors are low grade, indolent (slow growing), genetically stable, and devoid of p53 (*TP53*) mutations, mostly presenting at early stage. They include low-grade serous carcinoma, low-grade endometrioid carcinoma, clear-cell carcinoma, mucinous carcinoma, and malignant Brenner tumors [7,36]. Type I tumors are associated with wild-type p53 (*TP53*), but often contain mutations in genes such as *KRAS*, *BRAF, PTEN*, and β-catenin [7,36,39]. In contrast, Type II tumors are high grade, inherently aggressive, genetically unstable, typically harboring p53 (*TP53*) mutations, and presenting at advanced stage [40,41]. Included in this group are high-grade serous carcinoma (HGSC), high-grade endometrioid carcinoma, undifferentiated carcinomas, and malignant mixed-mesodermal tumors (MMMT; carcinosarcoma) [7,36]. Low-grade cancers resemble normal cells cytologically, whereas high-grade cancers show variation in cellular size and shape, large and irregular nuclei, more frequent mitoses, and loss of polarity [42]. 

Unlike non-epithelial ovarian tumors, EOCs are diagnosed predominantly at advanced stage (stage III or IV: 60–80%) with widespread metastases throughout the peritoneal cavity, which is associated with high mortality [11,14,43]. In contrast, a smaller fraction of EOCs (20–40% of EOCs) are diagnosed at early stage (stage I or II) [8,11,14]. A recent, large comprehensive histotype analysis of 28,118 cases of EOCs—diagnosed in 2004–2014, drawn from the U.S. Surveillance, Epidemiology, and End Results (SEER) cancer registry data—indicates that 39.2% (11,009/28,118) of EOCs are diagnosed in early stages (stage I and II) and 60.8% (17,109/28,118) of EOCs in advanced stages (stage III and IV) [14]. These early-stage tumors are dominated by type I (low-grade) tumors (61.1%: 6728/11,009) [14] (~85%) [11]: low-grade serous, endometrioid, mucinous, and clear-cell carcinomas. Two-thirds (75.6%: 6728/8900) of the type I, low-grade carcinomas are diagnosed in early stages [14,44]. Generally, the type I tumors are clinically indolent tumors, and thus a relatively minor contributor to ovarian cancer deaths [9,45]. Still, type I tumors account for 18.6% (2235/12,045) of EOC deaths [14]. In contrast, while constituting a minor fraction (38.9%: 4281/11,009) of early-stage EOCs, type II tumors (HGSC and carcinosarcoma) account for the vast majority (87.3%: 14,937/17,109) of advanced-stage EOCs and for most EOC deaths (81.4%: 9810/12,045) [14]. Among the type II tumors, HGSC is the predominant type. HGSC alone accounts for 81.1% (13,898/17,109) of all advanced-stage EOCs and is responsible for nearly three-quarters (73.9%: 8900/12,045) of all EOC deaths [14].

Overall, high-grade serous carcinoma (HGSC), also known as high-grade serous ovarian cancer, is estimated to be 50–60% of all ovarian malignancies [10,11]. Moreover, HGSC accounts for a large majority (63.4%: 7837/28,118) of all ovarian carcinomas, and advanced-stage HGSC represents nearly a half (49.4%: 13,898/28,118) of all EOCs [14]. Hence, when the term “ovarian cancer” or “epithelial ovarian cancer” is used without specific subtype elaboration, most often, it refers to “high-grade serous ovarian cancer (HGSC).”

#### High-Grade Serous Ovarian Cancer (High-Grade Serous Carcinoma: HGSC)

**Figure 1:**
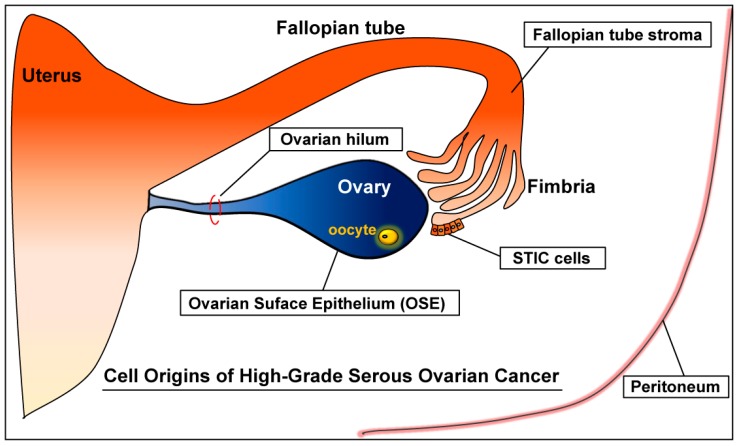
Cells of origin for high-grade serous ovarian cancer (HGSC).

High-grade serous ovarian cancer refers to the HGSC arising from the ovary, fallopian tube, or peritoneum [9,46] (Figure 1). Yet HGSC (high-grade serous ovarian cancer) should be distinguished from (high-grade) endometrial serous carcinoma. Serous carcinoma arising from the endometrium is also classified as high-grade serous carcinoma, but of uterine origin; hence, it is an endometrial cancer, not an ovarian cancer [47]. This uterine cancer is also commonly called uterine papillary serous carcinoma (UPSC). To distinguish from (high-grade) endometrial serous carcinoma (UPSC), the HGSC of primary ovarian, tubal, or peritoneal malignancy is also called high-grade pelvic (nonuterine) serous carcinoma [48]. HGSC accounts for more than 60% of epithelial ovarian cancers and over 70% of all ovarian cancer deaths [10,14,41]. Thus, HGSC is not only the most common, but also deadliest ovarian cancer [41].

The primary reason for the high mortality associated with HGSC is its diagnosis predominantly at advanced stage [11,14,45,49]. Generally, despite overall high mortality, ovarian cancer has a much better prognosis when diagnosed in the early stages [50]. When ovarian cancer is diagnosed in stage I, and when tumors are still localized to the ovary (15–20% of all cases [2]), the five-year survival rate is 92.3% after treatment with conventional surgery and platinum-based chemotherapy [50]. The five-year survival is still 74.5%, even when the disease has spread to the pelvis in stage II. For advanced-stage ovarian cancer, however, the five-year survival falls to 29.2% [50]. These observation suggest that early detection of ovarian cancer would improve treatment outcomes and survival [51]. Challenging this seemingly obvious notion, however, is the fact that most of early-stage diagnoses are indolent tumors [8,11,44]. The vast majority (>80%) of ovarian cancers detected in stage I are non-epithelial tumors and low-grade epithelial tumors, which are mostly indolent, portending a favorable prognosis [8,11,44]. In contrast, HGSC, which accounts for the majority of ovarian cancer deaths, is not frequently detected in early stages (<25%), with most cases of HGSC being diagnosed in stage III or IV (>75%) [11,14,45,49]. As HGSC account for more than 80% of advanced-stage (III–IV) ovarian cancers and over 70% of ovarian cancer deaths, effective early detection would require detection of a greater fraction of HGSC in early stages prior to distant metastasis [14,45].

HGSC can be detected in early stages. In the Normal Risk Ovarian Cancer Screening Study (NROSS) in the US with more than 5000 women [52], 21 operations were prompted by the screening strategy to detect 13 ovarian cancers with 9 in early stage (I/II). Two of the 9 were borderline, and 7 were invasive. Of the 7 invasive early-stage ovarian cancers detected, 6 were high-grade serous or endometrioid. While the numbers are small, these updated results from the NROSS suggest that HGSC can be detected in the early stage, although there is clearly room to improve the sensitivity of currently-available serum biomarkers and imaging techniques [52].

In addition, early-stage diagnosis of HGSC is not rare. In the large study of the U.S SEER data, HGSC accounts for more than a third (35.8%: 3939/11,009) of early-stage (I and II) EOCs: 19.1% (88/4621) in stage IA/IB (localized) and 47.9% (3057/6388) in stage IC/II (regional) [14]. This study also shows that patients with advanced-stage HGSC have a poor prognosis: 32.1% for 5-year survival and 15% for 10-year survival. In contrast, in patients diagnosed with early-stage HGSC, survival rates improve to 71.4% (5-year) and 53% (10-year), respectively. These improvements in survival among patients with early-stage HGSCs are fairly comparable to survival rates in patients with early-stage type I (indolent) tumors (80.4% for 5-year and 68.0% for 10-year survival). This suggests that effective early-stage detections of HGSC could improve overall patient survival in ovarian cancer.

By far, the largest screening trial of ovarian cancer has been a randomized clinical trial from the United Kingdom Collaborative Trial of Ovarian Cancer Screening (UKCTOCS) [53], in which a total of 202,638 postmenopausal women ages 50 to 74 had been followed for a median of 11.1 years and evaluated for ovarian cancer mortality, after a randomization into a control (no screening) group and two screening groups: (i) serum CA125 and (ii) transvaginal ultrasound (TVUS). The primary analysis shows no significant reduction in ovarian cancer mortality, though ovarian mortality rates are reduced in screening groups by 15% (CA125) and 11% (TVUS), compared with no screening group. There is no difference in all-cause mortality between screening and control groups. Curiously, a secondary analysis, after exclusion of deaths in years 0–7, produces a mortality reduction of 21% in the CA125 group (*p* = 0.021; 95% confidence interval: −2 to 40%). Overall, the results are in line with those from previous ovarian cancer screening trials, which have not shown a significant decrease in ovarian cancer mortality [44,51,54,55,56].

As HGSC is responsible for more than 70% of ovarian cancer deaths and over 80% of advanced-stage ovarian cancer diagnoses, key to effective ovarian cancer screening is an effective detection of early-stage HGSC. This appears to be challenging. When women with symptoms were promptly diagnosed by CA125 blood test and TVUS, seven of nine (78%) HGSC cases were already in advanced stages (III–IV) with two HGSC cases (22%) in early stages [49], illustrating the challenge of diagnosing HGSC at an early stage with the currently available detection methods.

In principle, the goal of cancer screening is straightforward: detection of cancer at an early, curable stage to reduce cancer mortality and improve patient survival. In practice, however, it is profoundly challenging to detect eventual life-threatening malignancies in their early stages among asymptomatic individuals, who may be at average risk or genetically high risk. Considering the relatively low incidence of ovarian cancer (lifetime risk: 1.3% for ovarian cancer vs. e.g., 12.4% for breast cancer), an effective screening test needs to be equipped with high sensitivity as well as robust specificity. Presently, less than a quarter (22.1%: 3939/17,837) of HGSCs are diagnosed in early stages, while more than three-quarters (77.9%: 13,898/17,837) of HGSCs are not diagnosed until advanced stage [14]. The future success of ovarian cancer screening will therefore depend on how effectively these advanced-stage diagnoses of HGSC could be shifted to early-stage diagnoses.

Achieving effective early-stage detection of HGSC will also require a better understanding of the mechanism of HGSC, particularly early mechanisms, including the cell(s) of origin, cancer initiation and development, early progression, and metastatic transition. As ovarian cancer, particularly HGSC, presents mostly as an advanced-stage disease with widespread peritoneal metastases, it would often not be clear, at the time of diagnosis, where the tumors actually originate in a given patient. Nevertheless, ovarian cancer, including HGSC, was presumed, by convention, to originate in the ovary [18,19,57].

## 2. Origins of High-Grade Serous Ovarian Cancer (HGSC)

### 2.1. Ovary

#### Ovarian Surface Epithelium (OSE)

Epithelial ovarian cancer has been thought to arise from epithelium in the ovary—the ovarian surface epithelium (OSE). Most ovarian cancer patients present with advanced-stage disease, in which tumors are found in the ovary and other peritoneal tissues, including the fallopian tube, mesentery, omentum, and diaphragm. In some patients, however, tumors are confined to the ovary. Though observed in different patients, viewing these tumors as different phases of the same malignancy, ovarian cancer with advanced-stage disease was assumed to have originated in the ovary [18,19,57]. The vast majority of advanced-stage ovarian cancers are high-grade serous carcinoma (HGSC). Hence, HGSC is also thought to arise from the ovary [18,58]. In the ovary, epithelial cells reside in the OSE. During ovulation, as the mature ovarian follicle ruptures and releases the egg, it also causes a local breakage of the OSE at the ruptured site. As the ruptured follicle differentiates into a corpus luteum, the damaged OSE may invaginate during the repair process. The inward movement of the damaged OSE toward the ovarian cortex (stroma) could result in the formation of a cyst with an epithelial lining inside (termed an “inclusion cyst”) [57,59,60]. Alternatively, it was suggested that inclusion cysts could form, without ovulation, as a result of an interaction between the OSE and the ovarian stroma [57]. It has been theorized that OSE and cortical inclusion cysts derived from the OSE may be the origin of all epithelial ovarian cancers [18,19,57,58].

##### OSE: Developmental View

The OSE is a single layer of squamous (flattened) epithelial cells derived from the coelomic epithelium, which lines the body cavity (coelom) of a developing embryo [59]. In the peritoneal cavity, the coelomic epithelium differentiates into mesothelium, the epithelial cells lining the peritoneum [45]. The part of the coelomic epithelium overlying the gonadal ridge, in which the ovary forms, differentiates into the OSE [59,61]. The OSE is thus a type of mesothelium covering the ovary; hence, the OSE is also known as ovarian mesothelium (OM) [59]. Interestingly, though both tissues are histologically mesothelium, the peritoneal mesothelium and the ovarian mesothelium (OSE) give rise to distinct malignancies. When the mesothelium lining the peritoneum undergoes a malignant transformation, the resulting tumor is called (peritoneal) mesothelioma [45]. On the other hand, transformation of the OSE (ovarian mesothelium) leads to ovarian carcinoma.

The coelomic epithelium also invaginates at the upper lateral part of the gonadal ridge, forming the Müllerian ducts [19,59]. The Müllerian ducts give rise to the epithelia of the fallopian tube, uterine endometrium, and endocervix (the upper part of the cervix) [19,59]. Thus, the peritoneal mesothelium, the ovarian surface epithelium (ovarian mesothelium), and the epithelium derived from the Müllerian ducts share the same embryonic origin: the coelomic epithelium [19,59]. Despite this, the peritoneal and ovarian mesothelium and the Müllerian duct-derived epithelium are phenotypically different [19]. The normal epithelial cells in the fallopian tube, uterus, and endocervix are columnar (tall) epithelium, whereas the peritoneal and ovarian mesothelium are flat-to-cuboidal epithelial cells [59]. Unlike the peritoneal mesothelium, the OSE (ovarian mesothelium) is prone to metaplasia (replacement of one mature cell type with another mature cell type) [19,57]. The OSE tends to spontaneously undergo metaplasia to resemble the normal epithelium of the fallopian tube, uterus, and endocervix [19,57]. This metaplastic capability of OSE to Müllerian epithelium, when combined with gene mutations, could prompt the formation of ovarian carcinomas bearing a morphological similarity to the normal epithelium of the fallopian tube, uterus, and endocervix [62]. Hence, though not naturally resembling the cellular morphology (Müllerian epithelium) of ovarian carcinomas, the OSE appears to have the capacity to transform into ovarian carcinomas bearing the morphology of Müllerian epithelium.

##### OSE: Mouse Models

Mouse studies have provided abundant evidence that the OSE can transform into ovarian carcinomas resembling the morphology of Müllerian epithelium [62,63,64,65,66,67,68] (Table 1). In an allograft study, individual expression of *Hoxa9*, *10*, and *11* in the transformed mouse OSE results in ovarian carcinomas resembling serous, endometrioid, and mucinous ovarian carcinoma, respectively, when these *Hoxa*-expressing OSE cells are injected into mice [62]. The OSE has also been genetically modified by targeted mutations in genetically engineered mouse models. When *Pten* is inactivated and simultaneously *Kras* ^G12D^ mutant is expressed specifically in the OSE by a delivery of adenovirus-cre to the ovarian bursa, these mice develop endometrioid ovarian carcinoma with peritoneal metastases [66]. Adenovirus-cre-mediated inactivation of *Pten* and *Apc* in the murine OSE leads to endometrioid ovarian carcinoma [68]. When *Pten* deletion and *Kras*^G12D^ expression are induced by *Amhr2* ^cre/+^, the OSE transforms to produce low-grade serous carcinoma [67]. With an addition of p53 (*Trp53*) ^R172H/+^ mutant expression to *Pten* deletion and *Kras*^G12D^ expression, these mice form mucinous carcinomas from the OSE, coexisting with cells of serous features [69].

Ovarian carcinomas histopathologically resembling human HGSC can also arise from the OSE [65,72,74] (Table 1). Mutations in the p53 gene (*TP53* in humans; *Trp53* in mice) is the most common genetic event observed in human HGSC [41]. Inactivation of *p53* (*Trp53*) and *Rb1* in the OSE, via an intrabursal injection of recombinant adenovirus cre, leads to metastatic serous ovarian carcinoma [65]. In this model, nearly all mice (97%: 33/34) develop ovarian tumors which may be histologically classified as serous or undifferentiated carcinoma. Though the majority of these ovarian tumors appear to remain at stage I, 27% (9/33) of the mice exhibit peritoneal metastases with accompanying ascites. Lung metastases are noted in 18% (6/33) of the mice, and 6% (one mouse) develop liver metastases. This reported phenotype, however, was not reproducible in another independent study [70,71]. In this second study, the same experimental approach produced leiomyosarcoma in the ovary—a smooth muscle tumor—instead of the reported metastatic (high-grade) serous ovarian carcinoma [70]. In another mouse study, adenovirus cre was delivered inside the bursa to delete or express in the OSE the following genes: (i) inactivation of *Rb1*, *p53*, and *Brca1*; (ii) expression of a *p53* mutant (p53^R172H^) with *Rb1* and *Brca1/2* deletion [72]. These mice developed ovarian tumors with high-grade serous morphology, ranging from stage I to IV [72]. Examined closely, some of these ovarian tumors remained at stage I or II (29%: 21–32%), lacking metastatic capability. Peritoneal metastases were not common: 16% (0–26%) of the mice were at stage III. Without accompanying peritoneal metastasis, liver, lung, or plural metastases were observed in 17% (0–25%) of the mice.

In another model, loss of *Lkb1* and *Pten* in the OSE leads to ovarian HGSC (100%: 12/12), but with seemingly weak metastatic potential: ascites noted in 25% (3/12) of the mice [74]. In addition, in a transgenic mouse model expressing SV40 large T antigen (TAg) in the OSE, ~50% of the mice produce metastatic ovarian carcinoma of serous morphology [63]. Also, ovarian carcinoma with peritoneal metastasis is formed in an allograft model, in which mice are injected with the OSE harboring p53-null mutation and any two oncogenic mutations of *Myc*, *Kras*^G12D^, and *Akt* [64].

Evidently, the mouse OSE, incited by gene mutations, is capable of transforming into an ovarian carcinoma histopathologically resembling human HGSC. Yet, the OSE-derived HGSCs from mouse models seem to exhibit weak metastatic potential. Conversely, human HGSC appears to be inherently aggressive, and to be capable of metastasis from a relatively early stage of development [8,9]. It is possible that additional mutations could enable aggressiveness of OSE-derived murine HGSCs. However, a number of mouse models, exhibiting generally weak or deficient metastatic ability of OSE-derived HGSCs, also suggest another possibility: though likely serving as the origin for some HGSCs, the OSE may not be the only primary site from which human HGSC arises [9,73,75,76,77,78,79].

### 2.2. Fallopian Tube

#### 2.2.1. Serous Tubal Intraepithelial Carcinoma (STIC)

Beginning in the late 1990s, the fallopian tube has emerged as another likely site of origin for high-grade serous carcinoma (HGSC) [9,76,80,81,82]. This insight came from studies of women carrying germline *BRCA1* or *2* mutations, which make these women more prone to malignancies, particularly ovarian cancer as well as breast cancer [83,84,85]. In these *BRCA* carriers, the lifetime risk of ovarian cancer ranges from 40–60% for *BRCA1* and 10–30% for *BRCA2*, respectively [2,76,84,86]. The most common type of ovarian cancer noted in these *BRCA1* or *2* carriers is HGSC [87,88]. When *BRCA* mutations were first recognized as conferring high risk for ovarian cancer, ovarian cancer was believed to arise solely from the ovary. Further evaluation, however, demonstrated that *BRCA* carriers were also susceptible to peritoneal and fallopian tube malignancies, as well as ovarian cancer [89,90,91,92,93,94]. Hence, the standard risk-reducing prophylactic procedure has become surgical removal of the ovaries and fallopian tubes (bilateral salpingo-oophorectomy), preferably by age 40 [84,95].

An early observation implicating the fallopian tube as a site of origin for HGSC came from a study of the fallopian tubes, prophylactically removed by surgery, from high-risk women including *BRCA1* carriers [80]. In this study, half of the high-risk women (6/12) exhibited dysplasia (preneoplastic change) in the epithelium of their fallopian tubes [80]. In contrast, little abnormality was found in the prophylactically-removed ovaries from *BRCA1* carriers [96,97]. These observations prompted more extensive histopathological examinations of the ovaries and fallopian tubes prophylactically removed from germline *BRCA*-mutation-carrying women [96]. These studies had led to the discovery of a potential premalignant lesion in the epithelium of the distal fallopian tube (fimbria), termed “serous tubal intraepithelial carcinoma (STIC)” [98,99].

##### STIC: Clinical and Molecular Observations

STIC is a noninvasive premalignant lesion with malignant cellular features [45,48], including enlarged nuclei, dark staining of the nucleus (hyperchromasia), coarse chromatin aggregates, and prominent nucleoli, which are also characteristic histopathological features of HGSC [9]. STIC is closely associated with HGSC. STIC lesions are found in prophylactically removed fallopian tubes from asymptomatic germline *BRCA*-mutation carriers (0–11.5%) [100,101,102,103]. In addition, STICs are also identified in the fallopian tubes of patients with sporadic (nonhereditary) HGSC (21–59%) [45,48,104,105,106], as well as germline-*BRCA*-mutation-positive women with hereditary HGSC (3–31%) [107,108]. Most STICs exhibit robust immunostaining of p53 [109,110] and harbor p53 mutations [111] (collectively termed the “p53 signature”). The p53 signature refers to benign-appearing secretory cells in the distal fallopian tube that exhibit intense nuclear p53 (TP53) staining, positive γ-H2AX staining (indicative of DNA damage), and lack of Ki-67 (MIB-1) staining (indicative of minimal proliferative activity) [98,109,112,113]. As p53 signatures lack histological features of STIC, p53 signatures are considered earlier lesions preceding STICs [101,114]. Besides the histopathological resemblance and the association of its occurrence to HGSC, STIC lesions also exhibit genomic instability, a characteristic genomic feature of HGSC, indicating genetic similarity to HGSC [114,115]. In addition, genomic analysis of STIC lesions, fallopian tube tumors, ovarian tumors, and peritoneal metastases from the same patients reveals an evolutionary relationship, suggesting that STICs and p53 signatures are likely early events in the progression of HGSC [114,116]. Furthermore, the evolutionary analysis of genetic changes observed in these various tumor tissues has identified alterations in *BRCA1*, *BRCA2*, *TP53*, and *PTEN* as critical early events in the initiation of STICs and subsequent development of HGSC [114]. Also, gene-expression profile of HGSC exhibits a greater similarity to that of the fallopian tube epithelium than to the ovarian surface epithelium, suggesting a fallopian tube origin of HGSC [117]. Together, these observations have led to the hypothesis that the fallopian tube STIC is a precursor of HGSC arising from the ovary, fallopian tube, or peritoneum [9,76,118,119].

According to this hypothesis, STICs could develop into invasive tumors (HGSC) in the fallopian tube, and the fallopian tube HGSCs then spread to the ovary and peritoneal cavity. Alternatively, STICs could shed and implant on the surface of the ovary or peritoneum where they could progress to HGSC [9,120].

##### STIC: Mouse Models

Crucial to this hypothesis is the demonstration of a cause-effect relationship between STIC and HGSC: i.e., whether STIC can transform into malignant and metastatic HGSC. Partial evidence has come from studies with genetically engineered mice, in which the fallopian tube epithelium is preferentially, or in combination with other tissues, targeted with gene mutations [78,79,121,122,123,124,125] (Table 2). In one mouse study, expression of mutant p53 (*Trp53*) and inactivation of *Pten* and *Brca1/2*, together, generated STICs (83.9%: 26/31 mice) as well as HGSCs in the ovary (73.1%: 19/26) and peritoneum (73.1%: 19/26) [78]. In another study, STIC formed in 35% (28/80) of mice harboring the inactivation of four genes (*Brca1, p53, Rb1,* and *Nf1*: 48 mice) or three genes (*Brca1, p53,* and *Rb1*: 29 mice; *Brca1, p53,* and *Nf1*: 3 mice) in the fallopian tube epithelium [79]. Also, in this study, more than two-thirds of the mice (68.8%: 55/80) formed fallopian tube tumors (STIC, early-stage HGSC, and HGSC); some of these mice also developed ovarian tumors (38.2%: 21/55) or peritoneal tumors (12.7%: 7/55) or ascites (12.7%: 7/55), or a combination of the three [79]. In the same study [79], inactivation of *Brca1*, *p53*, and *Pten* in the fallopian tube epithelium also produced STIC or early-stage HGSC or both or HGSC in the fallopian tube in 90% of mice: STIC (40%: 4/10 mice); early-stage HGSC (60%: 6/10); and HGSC (20%: 2/10). In these mouse models [78,79], as the gene mutations occur in the fallopian tube epithelium—but not in the ovary or peritoneum—ovarian and peritoneal HGSCs are likely tumors resulting from STICs formed in the fallopian tube.

Overall, these mouse models, targeting fallopian tube epithelium with gene mutations, develop STICs and HGSCs which closely recapitulate many of clinical and molecular features of STIC and HGSC in humans. Also, notably, *Brca1* or *2* inactivation appears to be necessary for STIC to advance to HGSC in mice [78]. These mouse models therefore support a notion that STIC can be a precursor lesion for HGSC in genetically high-risk women carrying germline *BRCA1/2* mutations.

It remains unclear, however, whether STIC could also serve as a precursor for sporadic (nonhereditary) HGSCs, which account for 85–90% of all HGSC cases [2,76]. In mouse models without *Brca1* or *2* mutation, *p53* mutation and *Pten* deletion together can produce STIC lesions in the fallopian tube epithelium (67%: 4/6 mice) (Table 2) [78]. Yet these STICs do not progress to invasive or metastatic HGSCs in the ovary or peritoneal cavity [78]. In another study, expression of the SV40 large T antigen (TAg), driven by the oviductal glycoprotein 1 (*Ovgp1*) promoter, also results in STIC lesions in the fallopian tube epithelium along with adenocarcinoma in the ovary in some mice [123]. As *Ovgp1* is highly expressed in the fallopian tube and TAg expression is not detected in the ovarian surface epithelium, this ovarian carcinoma is presumed to have resulted from the spread and transformation of fallopian tube STICs in the ovary [123]. Nevertheless, these STIC lesions do not advance to peritoneal HGSC [123]. Thus, these mouse studies suggest that many STIC lesions may not progress to invasive, and more critically, metastatic malignancies [78,79,123]. Studies of human HGSCs also note that most STIC lesions likely do not advance to metastatic HGSC, and may thus be classified as low grade [98,126]. Hence, it remains to be elucidated whether STIC could be a bona fide precursor lesion for HGSC in women in the general population who are at average risk, and yet who account for most cases of HGSC.

##### STIC: Clinical Significance

Overall, though existence of STIC and its association to HGSC are extensively described in human and mouse studies, the clinical significance of STIC remains uncertain [127]. In the early studies, in which the fallopian tube was first proposed as a potential primary origin of HGSC, tubal dysplastic lesions, later termed STICs, were reported in 37% and 50% of high-risk women whose fallopian tubes were prophylactically removed [80,118] (Table 3). In most clinical studies, however, including ones with larger sample sizes, the prevalence of STICs (or occult tubal carcinomas) in high-risk women generally varies from 0–11.5% [100,101,102,103,128,129,130,131,132,133,134,135,136,137,138,139] (Table 3). It is unknown what proportion of the STICs would progress to malignant and metastatic HGSC. It is possible that HGSC could also develop from precursors distinct from STICs. Human and murine studies have indicated that metastatic HGSC can arise from the fallopian tube without evidence of STIC [77,79,120].

Also, STIC lesions may not be unique to HGSC. Though STICs are associated chiefly with HGSC, they are not exclusive to HGSCs of the ovary, fallopian tube, and peritoneum. STICs are also found in the fallopian tubes of patients with (high-grade) endometrial serous carcinoma (8–23%) [47,106,143,144], as well as ones from patients with nonserous endometrial carcinoma or endometrial hyperplasia (1.1%) [140] (Table 3). STICs are also found in the fallopian tubes of 3% (2/64) of women undergoing surgery not related to ovarian, tubal, or peritoneal malignancy [101]. In addition, STIC lesions are not found in a large fraction of clinical HGSC cases. In advanced human HGSC, the occurrence of STIC ranges from 21–59% in sporadic HGSC [45,48,104,105,106,141] and 3–31% in (germline-*BRCA*-mutation-positive) hereditary HGSC [107,108]. These findings suggest that a significant number of HGSCs may derive from precursors independent of STICs [36,108]. Also, p53 (*TP53*) signatures, another potential precursor lesions in the fallopian tube closely associated with STICs, is not unique to high-risk women, but is also commonly seen in women who are at low risk of ovarian cancer [101,109]. The p53 (*TP53*) signatures were present in the fallopian tubes of 19% (12/64) and 33% (19/58) of women who are at average risk of ovarian cancer, compared with 11% (19/176) and 24% (10/41) of high-risk women undergoing risk-reducing salpingo-oophorectomy (RRSO) [101,109]. The fact that STIC and p53 (*TP53*) signatures are not unique to HGSC and HGSC can arise without evidence of STIC suggests that there may exist additional, yet undiscovered, precursor lesions for HGSC [36]. Given the highly aggressive nature of HGSC, the existence of novel precursor lesions would not be inconceivable.

STIC is evidently a risk factor for HGSC. Yet, more causal evidence would be needed to affirm that STIC is a bona fide precursor lesion for hereditary (10–15%) and sporadic (85–90%) HGSCs in women. The key issue is whether STIC, a lesion of noninvasive neoplastic cells, could evolve into an invasive tumor and advance to aggressive metastatic HGSC. In the aforementioned mouse models [78,79], though some fallopian tube STICs lead to ovarian and peritoneal HGSCs, the extent and spectrum of metastases do not appear to fully match the clinical metastases observed in human HGSC.

Regarding the fallopian tube as an origin of HGSC, currently available data indicate that STIC likely serves as a premalignant lesion that could develop into metastatic HGSC in high-risk women carrying *germline BRCA1/2* mutations [145]. The overall fraction of ovarian cancers that originate in the fallopian tube is not known. In one study examining specimens from risk-reducing salpingo-oophorectomy (RRSO), microscopic HGSC was identified in the fallopian tube in six (4.5%) of 133 *BRCA1/2* carriers [113]. Four of the HGSCs were confined to the fallopian tube, one to the ovary only, and one was identified from peritoneal washings only. In contrast, when early-stage (I and II) HGSCs (14/131) from *BRCA1/2* carriers were examined, the majority of early-stage HGSCs (78.6%: 11/14) were diagnosed as ovarian primaries, while three cases (21.4%: 3/14) as fallopian tube primaries [113]. These results suggest that the fallopian tube may be the primary site of origin of HGSC, but the ovary is a preferred site of tumor growth and progression in high-risk women. Temptingly, this notion might be extended to HGSC in the general population, but there is not yet sufficient causal evidence [127]. 

It seems logical to predict that a precursor lesion such as STIC, which exhibits malignant morphological and genetic features, would gradually progress and eventually manifest as a full-blown metastatic malignancy [146]. However, it is also increasingly recognized that many of microscopically cancerous precursor or early-tumor lesions may not proceed to clinical, lethal malignancies [81,147,148,149,150]. Thus, rigorous evaluations of the causal relationship between STIC and metastatic HGSC would be critical for establishing STIC as an origin of metastatic HGSC.

##### Insights from Ductal Carcinoma In Situ (DCIS) in Breast Cancer

Ductal carcinoma in situ (DCIS), a microscopic malignancy confined to the breast ductal epithelium without invasion of the duct wall, is widely believed to be a precursor or premalignant lesion for all breast cancer malignancies [151]. In 2018, DCIS was estimated to be 24% (63,960) of newly diagnosed breast tumors (266,120) [152]. Since the 1970s, when screening mammography was introduced, detection of DCIS had risen by 700% from 1976 to 2008 (7 DCIS/100,000 women in 1976 and 56 in 2008) [153]. An additional 573,000 cases of DCIS were estimated to be diagnosed during the three decades. If DCIS is a true premalignant lesion for invasive and lethal breast cancer, more cases of DCIS should lead to a proportional decline in the incidence of advanced-stage breast cancer. However, late-stage breast cancer cases had fallen by only 8% during the same period (102/100,000 women in 1976 and 94 in 2008; diagnosis of additional 573,000 cases of DCIS vs. an estimated decrease of 67,000 cases of late-stage breast cancer during this period) [153]. These clinical observations suggest that though some DCIS can advance to invasive breast cancer, the vast majority of DCIS lesions are unlikely to progress to metastatic, life-threatening malignancies [154].

In an autopsy study in which women died from causes other than breast cancer, DCIS was identified in about 30% of women between the ages of 40 and 54 years [155]. As one in eight women (12.4%) develops breast cancer during her lifetime in the U.S. [156], this common occurrence of DCIS in the general population also bolsters the notion that many DCIS lesions do not lead to malignant breast cancers [155]. Some DCIS lesions do proceed to invasive, metastatic breast cancer [157]. It is unknown, however, which DCIS would advance to invasive, metastatic cancer and which would not; the estimates vary widely from 0–50% [154,157,158]. For this reason, though noninvasive, DCIS is still regarded as breast cancer (stage 0) and treated as aggressively as invasive breast cancer with surgery and hormonal therapy [154]. There is a growing perception that the rise of DCIS diagnoses has led to overdiagnosis and overtreatment [147,159].

Reflecting an increasing awareness of the overdiagnosis and overtreatment, there has also been a growing level of recognition that many of premalignant lesions, albeit histopathologically classified as cancer, often do not progress to invasive or metastatic tumors [81,147,148]. Hence, it has been proposed that premalignant lesions, such as ductal carcinoma in situ (DCIS), not be labeled as cancer or carcinoma [147]. Instead, they may be reclassified using a more appropriate term reflecting their indolent clinical behavior, such as “indolent lesions of epithelial origin (IDLE)” [147]. Also, appreciating the generally low malignant potential of most STICs, a new term, such as “low-grade serous tubal intraepithelial neoplasia,” was suggested in place of “STIC” [81].

Like DCIS, STIC is also a noninvasive tumor of epithelial origin [45,48]. Though not invasive, these STIC lesions possess malignant cellular features [98] as well as widespread genomic alterations, and are therefore considered preinvasive or premalignant lesions [114,115,116]. It is intuitive to assume that these lesions would eventually become invasive in the local tissue and ultimately spread to other parts of the body. Yet many microscopic cancers may not, though some do, proceed to clinical malignancy [149,150,160,161]. Current evidence suggests that some STIC lesions would be capable of progressing to invasive tumors leading to peritoneal metastases in the context of germline *BRCA*-mutation carriers [78,79]. Other STIC lesions may not undergo malignant transformation, remaining noninvasive [78,79,123]. Particularly critical, yet unknown, is the clinical significance of STIC in the general population where 85–90% of HGSC cases occur. The information on the prevalence of STICs in the general population is limited, but several studies suggest that it could vary from 0.8–3.1% [101,140,142], while the lifetime risk of ovarian cancer is 1.3% (one in 78 women) [50,162]. Like DCIS, most likely, some STICs would possess malignant potential, while more STICs would not.

Advances in technology would increasingly facilitate detection of these noninvasive yet potentially cancerous lesions, such as DCIS and STIC, in the general population as well as high-risk individuals. The challenge is to be able to predict how these potential precancerous lesions would behave in the course of tumor progression: would these precancerous lesions cause little harm or turn deadly if left untreated? As STIC has emerged as a potential precursor for human HGSC, it is crucial to elucidate the natural progression of STIC in the context of HGSC development at the molecular as well as biological levels. A deeper understanding of the natural history of STIC would help develop ways to clinically assess the malignant potential of STIC lesions.

#### 2.2.2. Fallopian Tube Stroma

Fallopian tube origin of HGSC is also supported by a serendipitous phenotype in a mouse model, in which *Dicer1* and *Pten* are inactivated in the fallopian tube [77] (Table 2). These *Dicer1-Pten* double-knockout (DKO) mice faithfully and reliably reproduce the clinical behavior of human HGSC with 100% penetrance [77]. In these mice, HGSC forms in the fallopian tube, and then spreads to envelop the ovaries, and also aggressively metastasizes throughout the peritoneal cavity. Peritoneal metastases occur preferentially to the omentum and diaphragm with widespread tumors in the mesentery and peritoneal membrane, invariably accompanied by ascites. All of the mice die from peritoneal metastases of fallopian tube HGSC (*n* = 24/24; 6.2–13 months of age; mean survival: 9.4 months) [77]. Though metastases are generally confined to the peritoneal cavity in these mice, occasionally, HGSC metastasizes to the lungs (stage IV), as well as the peritoneal cavity (stage III). Besides phenotypic and histopathologic resemblance, there are also significant correlations in gene expression between mouse and human HGSCs [77]. Moreover, these mouse HGSCs also exhibit widespread genomic instability resembling human HGSC (unpublished).

However, while modeling the clinical behavior of human HGSC, histopathologically classified as HGSC, exhibiting marked genomic disarray, and unmistakably stemming from the fallopian tube, the HGSC in this model does not appear to originate in the fallopian tube epithelium, but rather, in the fallopian tube stroma [77]. In this model, *Amhr2-cre* (*Amhr2*^cre/+^), in which the insertion of *cre* recombinase gene is targeted to an endogenous *Amhr2* locus, would direct the deletion of *Dicer1* and *Pten* specific to the fallopian tube stroma, not in the epithelium [163]. Accordingly, no histopathologic abnormality or STIC lesions were present in fallopian tube epithelium [77]. In this mouse model, in the absence of *Dicer1* and *Pten*, stromal stem cells residing in the fallopian tube [163] may transform into HGSC in the fallopian tube, leading to widespread peritoneal metastases as well as the ovarian metastasis. Thus, despite fallopian tube origin, the stromal origin of HGSC in this mouse model is at odds with the STIC hypothesis, which predicts that the fallopian epithelium, particularly distal tubal epithelium, is the primary cell of origin of metastatic HGSC in humans. There is yet no clinical evidence that human HGSC could originate in the stroma of the fallopian tube.

Also, the genetic relevance of *DICER1* and *PTEN* in human HGSC is not clear. Low expression of DICER1 is associated with advanced stages and reduced survival in human ovarian cancer (HGSC) [164], suggesting that DICER1 may function as a tumor suppressor. Yet the role of *DICER1* loss in ovarian cancer remains to be clarified. In human HGSC, homozygous deletion of *DICER1* (*DICER1*^−/−^) is extremely rare (0.3%: 1/316 patients) [41,165,166]. Relatively common, however, is a single-copy loss of *DICER1* (44%). Similarly, PTEN deletion is found in 38.9% of the cases for combined homozygous (6.6%) and heterozygous losses (32.3%) [41]. Though PI3K signaling is frequently activated in human HGSC, it is not clear how this partial loss of *PTEN* impacts the development of HGSC.

Thus, most cases of human HGSC are unlikely to occur as a direct consequence of loss of *DICER1* and *PTEN*. Rather, this mouse model reveals critical pathways—activated by loss of *Dicer1* and *Pten*—what are essential to the development of metastatic HGSC. Accordingly, this mouse HGSC reveals significant alterations of known critical pathways for HGSC [41], including PI3K signaling [77], FOXM1 signaling (unpublished), and homologous recombination (unpublished).

Despite many features in common between this mouse model and human HGSC—most notably, the striking clinical resemblance to human HGSC—the major limitation of this model is the precise cell origin of this murine HGSC: fallopian tube stroma. According to current understanding, it is difficult to envision that human HGSC, an epithelial malignancy, could originate, not in epithelium, but rather in stroma. In the mouse uterus, a fraction of stromal cells of *Amhr2* lineage, likely stromal stem cells, are capable of differentiating into epithelial cells during the endometrial regeneration after parturition [163]. Plausibly, stem cells residing in the fallopian tube stroma, in the absence of *Dicer1* and *Pten*, transform into HGSC. Whether this potential stromal-to-epithelial transition could occur during the development of human HGSC remains to be clarified. Nevertheless, it should also be acknowledged that the natural course of human HGSC—initiation, development, early progression, and ultimate peritoneal metastasis—remains poorly understood.

Rarely does an animal model manifest a full spectrum of clinical disease of a human disorder. If an animal model, however, develops a cancer that behaves like the human cancer with a nearly identical metastatic pattern, one could reason that the mouse and human malignancies likely share similar mechanisms of development and tumor progression. This mouse model could be a valuable tool in understanding the mechanisms underlying the development, early progression, and metastatic progression of human HGSC—and also serve as a useful preclinical model for evaluating new therapies.

#### 2.2.3. Ovarian Cancer Prevention: Salpingo-Oophorectomy vs. Salpingectomy

Over the last decade, clinical and mouse studies have bolstered the idea that the fallopian tube is the predominant origin of HGSC. This novel concept has spawned new thinking in ovarian cancer prevention in women at high genetic risk and also in the general population.

Salpingo-oophorectomy—surgical removal of the ovaries and fallopian tubes—is the standard preventive surgery recommended for germline *BRCA1/2* carriers, who are at high risk of ovarian cancer [9,85,167,168]. This prophylactic surgery has proven to be highly effective, as it reduces the risk of ovarian cancer by 72–96% [86,89,169], ovarian-cancer-specific mortality by 79–95% [86,170], and overall mortality by 60–66% [86,170]. Besides ovarian cancer protection, oophorectomy also significantly decreases the risk of breast cancer (50% risk reduction) in *BRCA1/2* carriers [84,86,89,170,171,172,173] (though some studies indicate no difference in breast cancer risk, or a selective risk reduction only among *BRCA2* carriers [174,175]).

Despite the proven benefits of risk and mortality reductions in ovarian and breast cancer, salpingo-oophorectomy has a major drawback of premature menopause, which hinders wider acceptance. Prophylactic surgery involves removal of the ovaries and fallopian tubes, and is recommended between the ages of 35 and 40 years, when women are premenopausal [84,95,168,176]. Consequently, these women undergo premature surgical menopause with an increased risk of experiencing postmenopausal symptoms, including hot flashes, sleep disturbance, mood changes, vaginal dryness, sexual dysfunction, cognitive decline, osteoporosis, and cardiovascular disease [177,178,179]. To avoid these adverse health outcomes, approximately 30–50% of *BRCA1* and *2* carriers choose not to undergo prophylactic salpingo-oophorectomy even after completion of child bearing [95,180,181,182,183,184,185].

With the emerging concept of the fallopian tube as the principal site of origin for HGSC, an alternative preventive approach has been proposed to mitigate the adverse consequences of ovary removal in women at high genetic risk. A two-step surgery has been proposed, in which salpingectomy (removal of the fallopian tubes) is performed early upon completion of childbearing, followed by subsequent oophorectomy (removal of the ovaries) at ages 40–45 for *BRCA1* and ages 45–50 for *BRCA2* carriers [180,186,187]. Although salpingectomy alone with ovary conservation could theoretically reduce the risk of HGSC, this approach warrants careful consideration. 

While the majority of HGSCs arise in the fallopian tube, it may not be that all originate there [113,139]. Thus, retaining the ovaries with salpingectomy alone would continue to pose ovarian cancer risk to some high-risk women until the completion of oophorectomy [180,188]. Another concern is a delay in oophorectomy. Breast cancer protection appears to occur when oophorectomy is performed before menopause in high-risk women [86,89,170,172,173]. Delaying ovary removal may diminish the benefit of breast cancer protection [176,188]. Ovarian cancer risk appears to be higher in salpingectomy alone than salpingo-oophorectomy in the general population [189,190]. Clinical trials are underway to evaluate the effectiveness of salpingectomy alone or salpingectomy followed by delayed oophorectomy in high-risk women [176,186]. Presently, bilateral salpingo-oophorectomy is still the recommended prophylactic surgery for high-risk women carrying germline *BRCA1* or *2* mutations [176].

This new paradigm of fallopian tube origin also presents the option for an opportunistic salpingectomy as an alternative preventive measure in the general population (who are at average risk) undergoing hysterectomy for benign disease or pelvic surgery [95,191,192,193]. In the United States, approximately 600,000 women undergo a hysterectomy for benign (uterine) disease [194]. About half (300,000) the women undergoing hysterectomy also opt for a prophylactic salpingo-oophorectomy for a variety of health reasons, including risk reduction of ovarian cancer; the other half choose to retain their ovaries and fallopian tubes to avert the adverse health consequences associated with removal of the ovaries [191,195]. As expected, the risk of developing ovarian and breast cancer is reduced (by 96% and 25%, respectively) in average-risk women undergoing hysterectomy and salpingo-oophorectomy, compared with women undergoing hysterectomy but conserving the ovaries [196]. However, adverse effects of ovary removal are also pronounced in these women. Beyond premature menopausal symptoms, ovary removal has also been associated, in some studies, with increased mortality in women with hysterectomy (28% increase in the risk of death for coronary heart disease, and 12% increase in overall mortality), compared with women whose ovaries are retained at the time of hysterectomy [195,196]. Observational studies suggest that ovary removal may do more harm than good in average-risk women in the general population [195].

Germline *BRCA1/2* carriers have high risk of both ovarian and breast cancer (lifetime risk: 10-60% for ovarian cancer [2,86]; 66–82% for breast cancer [84,85]). In these high-risk women, removal of the ovaries offers dual benefits: not only does it prevent ovarian cancer, it also significantly lowers breast cancer risk (by 47–64%) [84,86,171] and mortality (by 56–90%) [84,86,170]. The benefits of removing the ovaries (as well as the fallopian tubes), therefore, appear to be evident in these women at high genetic risk. In the general population, however, women are at average risk for ovarian and breast cancer (lifetime risk: 1.3% for ovarian cancer [50,162]; 12.4% for breast cancer [50,152]). In these average-risk women, though ovary removal appears to reduce the risk of breast cancer, it seems to have little impact on breast cancer mortality [195,196]. In contrast to a clear risk reduction in high-risk women, ovary removal seems to bring to average-risk women a relatively modest benefit in relation to breast cancer.

These average-risk women may benefit from fallopian tube removal alone and retaining the ovaries. Salpingectomy alone is shown to reduce the risk of ovarian cancer by 35–64% in the general population [189,190]. Conservation of the ovaries would improve quality of life by averting premature menopausal symptoms, and also extend overall survival by reducing ovary-loss-associated mortality. Hence, salpingectomy with ovary retention, in lieu of salpingo-oophorectomy, can be an option to these average-risk women undergoing hysterectomy or pelvic surgery [95,193].

In summary, in high-risk women, until further evidence is available, risk-reducing salpingo-oophorectomy (RRSO) is recommended by age 40 for *BRCA1* mutation carriers and by age 45 for *BRCA2* mutation carriers [95,176]. On the other hand, for women at average risk of ovarian and breast cancer, salpingectomy alone—and keeping the ovaries—may be a prophylactic option to consider when undergoing hysterectomy or pelvic surgery [193].

### 2.3. Other Potential Origins of HGSC

#### 2.3.1. Secondary Müllerian System

Epithelial ovarian cancer may also arise in the secondary Müllerian system [197], which refers to the presence of Müllerian epithelium (i.e., epithelium of the fallopian tube, uterus, and endocervix) outside the indigenous locations (i.e., the fallopian tube, uterus, and endocervix). The secondary Müllerian system includes endometriosis (endometrium-like tissue present outside the uterus), endosalpingiosis (fallopian tube-epithelium-like epithelium on or beneath the peritoneal surface), and the rete ovarii (Müllerian epithelium-resembling tubular structures near the ovarian hilum, a junctional area between the ovary and the fallopian tube). As HGSC resembles normal fallopian tube epithelium, the secondary Müllerian system could serve as an origin of HGSC [198]. After prophylactic removal of the ovaries, some of these ovary-deficient high-risk women develop HGSC in the peritoneum [89,199]. This primary peritoneal HGSC is believed to arise from endosalpingiosis [45] or Müllerian metaplasia of peritoneal mesothelium [76].

#### 2.3.2. Ovarian Hilum

In addition, the epithelium (particularly stem cells) lining the hilum, a junctional area between the ovary and the fallopian tube, has been suggested to be a cell of origin for HGSC [61,73]. In a mouse allograft study, epithelial cells in the mouse ovarian hilum that are also positive for stem-cell markers are isolated and cultured followed by inactivation of p53 (*Trp53*) and *Rb1* [73]. When injected into the peritoneal cavity of mice, these hilum-derived potential stem cells lacking p53 and *Rb1* are able to form HGSC in the ovary (*n* = 7/8 mice) [73]. However, the metastatic behavior of this HGSC does not seem to align with the typical metastatic pattern of human HGSC, which is characterized by widespread peritoneal metastases. Though five (71%) of the seven mice with ovarian HGSC also developed lung metastasis, there seem no other peritoneal tumors besides the tumors formed in the ovary [73]. While it is intriguing to see that hilum-derived, stem-cell-like epithelial cells lacking p53 and *Rb1* have potential to form serous carcinoma in the ovary, this serous tumor appears to lack peritoneal metastatic potential, a characteristic clinical feature of human HGSC [43].

## 3. Conclusions

Once it seemed obvious to think that all ovarian cancers, including high-grade serous ovarian cancer (HGSC), originated in the ovarian surface epithelium (OSE) [18,19,57]. Presently, it seems equally compelling to think that HGSC arises from serous tubal intraepithelial carcinoma (STIC) formed in the distal fallopian tube epithelium [9,46,200]. Both may be the cell of origin in different fractions of cases. Abundant clinical observations, genetic evidence, and increasing consensus in the field all point to STIC as the precursor lesion, particularly in women at increased genetic risk [9,76,114,116]. Yet, it is still imperative to rigorously establish a cause-effect relationship between STIC and clinical HGSC: i.e., whether STICs progress to malignant and metastatic HGSCs. Particularly, the clinical significance of STIC remains uncertain in women in the general population, who account for 85–90% of HGSC cases. Undoubtedly, STICs pose high risk for HGSC, and certain STIC lesions could transform into clinical HGSC. However, logical and intuitive as it may seem, many of precursor or premalignant lesions, despite consisting of microscopically and genetically cancerous cells, would not progress to lethal malignancies [147,149,150]. Hence, it is crucial to understand the natural progression of STIC lesions at the molecular and biological levels. In particular, the focus should be directed toward assessing the clinical consequences of STIC lesions: i.e., those likely to remain benign vs. those with the ability to undergo full malignant transformation, progressing to metastatic HGSC. A deeper and more comprehensive molecular characterization of STICs in different stages of HGSC may help predict or determine the malignant and metastatic potential of STICs. Also, as STICs do not account for all HGSCs, it is also important to search for other potential cell origins for HGSC. The fallopian tube stroma in mice, for instance, has the capability to develop metastatic HGSC which faithfully reproduces the clinical metastasis of human HGSC.

Determining the precise cell(s) of origin of HGSC is crucial for improving early-detection and prevention rates of ovarian cancer, and could offer insight into devising effective treatment against advanced ovarian cancer.

## Figures and Tables

**Table 1:** Mouse models of ovarian cancer: ovarian origins.

Targeted Genes	Promoter	Ovarian Tumor	Metastasis	Ascites	Ref.
p53, Myc, Kras ^G12D^; p53, Kras ^G12D^, Akt; p53, Akt, Myc	RCAS viral vector	Poorly differentiated or serous carcinoma (in nude mice)	Peritoneal lining, Omentum, Diaphragm, Liver, Pancreas, Intestines, Kidneys	Yes	[64]
p53, Rb1	Adenovirus cre	Serous carcinoma: 97% (33/34 mice)	Peritoneal: 27% (9/33) Lungs: 18% (6/33) Liver: 6% (1/33)	24% (8/33)	[65]
p53, Rb1; p53; Brca1, Rb1; p53, Rb1, Brca1	Adenovirus cre	Leiomyosarcoma: 100% (44/44)	No	27% (12/44)	[70]
p53, Brca1	Adenovirus cre	Leiomyosarcoma or high-grade sarcoma: 56% (23/41)	No	No	[71]
SV40 TAg	Amhr2 (MISIIR)	Serous carcinoma: 50% (18/36)	Peritoneal metastasis including omentum: ?%	Yes (?%)	[63]
Hox9; Hox10; Hox11	pCMV-Tag	HGSC (Hox9) Endometrioid (Hox10) Mucinous (Hox11) (in nude mice)	No description (ND)	ND	[62]
Pten, Apc	Adenovirus cre	Endometrioid carcinoma: 100% (29/29)	Peritoneal: 21% (6/29)	76% (22/29)	[68]
Pten, Kras ^G12D^	Adenovirus cre	Endometrioid carcinoma: ?% (?/9)	Peritoneal: ?% Lungs: 43%	Yes (?%)	[66]
Pten, Kras ^G12D^	Amhr2 ^cre/+^	Low-grade serous carcinoma:100% (8/8)	Omentum: 100% (8/8)	No	[67,69]
Rb1, p53, Brca1; Rb1, p53 ^R172H^, Brca1/2	Adenovirus cre	HGSC: Stage I and II: 29% (46/158; 21–32%)	Peritoneal: 16% (25/158; 0–26%) Liver or lung or pleural: 17% (28/158; 0–25%)	Yes (?%)	[72]
p53, Rb1 in the OSE hilum	Adenovirus cre	HGSC: 88% (7/8) (in NOD scid mice)	Lungs: 71% (5/7)	No	[73]
Pten, Kras ^G12D^, p53 ^R172H^	Amhr2 ^cre/+^	Mucinous carcinoma: 80% (8/10) Mucinous & serous: 100% (10/10)	Omentum: 100% (36/36)	No	[69]
Lkb1, Pten	Amhr2 ^cre/+^	HGSC: 100% (12/12)	No description	25% (3/12)	[74]
p53 ^R172H^, Pten	Amhr2 ^cre/+^	HGSC: 30% (15/50) mixed with granulosa cell tumor	Peritoneal HGSC: 100% (15/15); omentum, diaphragm, mesentery, peritoneal lining	80% (12/15)	[75]
p53 ^R172H^, Pten	Amhr2 ^cre/+^	Granulosa cell tumor: 70% (35/50)	Lungs: 53.3% (19/35)	No	[75]

?: information not described in the cited reference.

**Table 2:** Mouse models of ovarian cancer: fallopian tube origins.

Targeted Genes	Promoter	STIC	Fallopian Tube HGSC	Ovarian HGSC Metastasis	Peritoneal HGSC Metastasis	Ascites	Ref.
SV40 TAg	Ovgp1	–	Oviductal tumors (?%)	No ovarian tumor	No; Uterine tumor: 100% (26/26) Vaginal tumor: 62% (16/26)	No	[122]
- Monitoring of tumor development: 6–13 weeks of age							
Brca1, p53 ^R172H^, Pten	Pax8	100% (4/4)	No	25% (1/4)	25% (1/4): peritoneal mass	No	[78]
- Monitoring of tumor development: 5–7 weeks of age							
Brca2, p53 ^R172H^, Pten	Pax8	75% (9/12)	No	75% (9/12)	67% (8/12): peritoneal mass	No	[78]
- Monitoring of tumor development: 7–15 weeks of age							
p53 ^R172H^, Pten	Pax8	67% (4/6)	No	0% (0/6)	0% (0/6)	No	[78]
- Monitoring of tumor development: 19–38 weeks of age							
SV40 TAg	Ovgp1	Yes (?%)	No	Adeno-carcinoma (56%)	No	No	[123]
- Monitoring of tumor development: 8–10 weeks of age							
Brca1, p53, Rb1, Nf1	Ovgp1-iCreER	37.5% (18/48)	HGSC: 60% (29/48) MMMT: 25% (12/48)	HGSC or MMMT: 40% (19/48)	HGSC or MMMT: 13% (6/48)	13% (6/48)	[125]
- Monitoring of tumor development: 3.5–26 months of age							
Brca1, p53, Rb1	Ovgp1-iCreER	34.5% (10/29)	HGSC: 17% (5/29) MMMT: 7% (2/29)	0%	0%	0%	[125]
- Monitoring of tumor development: 3.5–26 months of age							
Brca1, p53, Nf1	Ovgp1-iCreER	0% (0/3)	HGSC: 67% (2/3) MMMT: 67% (2/2)	HGSC or MMMT: 100% (3/3)	HGSC or MMMT: 33% (1/3)	0%	[125]
- Monitoring of tumor development: 3.5–26 months of age							
Brca1, p53, Pten	Ovgp1-iCreER	40% (4/10)	HGSC: 80% (8/10) MMMT: 10% (1/10)	MMMT: 10% (1/10)	0%	10% (1/10)	[125]
- Monitoring of tumor development: 3–8 months of age							
Dicer1, Pten	Amhr2 ^cre/+^	No	100% (24/24)	100% (24/24)	100% (24/24): omentum, diaphragm, mesentery, peritoneal lining	100% (24/24)	[77]
- Survival range: 6.2–13 months of age (mean survival = 9.4 months; *n* = 24)							

MMMT: malignant mixed mesodermal tumor (carcinosarcoma); HGSC: high-grade serous carcinoma or high-grade serous ovarian cancer; p53: *Trp5*3; ?: information not described in the cited reference.

**Table 3:** Incidence of STIC in high-risk women and in the general population.

Sample Tissue	Population	Incidence of STIC or Occult Tubal Carcinoma	Number of Cases	Reference
Fallopian tubes from prophylactic salpingo-oophorectomy	High risk	50% (6)	12	Piek et al., 2001 [80]
		37% (16?)	44	Piek et al., 2003 [118]
		6.7% (4)	60	Colgan et al., 2001 [133]
		10% (3)	30	Leeper et al., 2002 [93]
		6% (4)	67	Powell et al., 2005 [137]
		8% (4)	50	Carcangiu et al., 2006 [131]
		3.8% (6)	159	Finch et al., 2006 [100]
		5.7% (7)	122	Callahan et al., 2007 [130]
		8.5% (15)	176	Shaw et al., 2009 [101]
		8.9% (4)	45	Hirst et al., 2009 [134]
		8.1% (9)	111	Powell et al., 2011 [128]
		8.5% (10)	117	Manchanda et al., 2011 [135]
		7.1% (16)	226	Mingels et al., 2012 [136]
		1.7% (5)	303	Reitsma et al., 2013 [138]
		4.2% (17)	405	Powell et al., 2013 [129]
		2.0% (12)	593	Wethington et al., 2013 [102]
		11.5% (9)	78	Cass et al., 2014 [132]
		2.6% (25)	966	Sherman et al., 2014 [139]
		0% (0)	111	Seidman et al., 2016 [140]
		5.6% (2)	36	Lee et al., 2017 [103]
Fallopian tubes from HGSC cases	High risk	30.8% (8)	26	Howitt et al., 2015 [108]
		3.3% (2)	60	Malmberg et al., 2016 [107]
Fallopian tubes from HGSC cases	General	47.6% (20)	42	Kindelberger et al., 2007 [104]
		58.5% (24)	41	Przybycin et al., 2010 [48]
		37.3% (19)	51	Seidman et al., 2011 [45]
		20.5% (8)	39	Tang et al., 2012 [106]
		38.3% (23)	60	Mingels et al., 2014 [141]
		38.2% (13)	34	Koc et al., 2014 [105]
		33.3% (6)	18	Malmberg et al., 2016 [107]
Fallopian tubes from non-ovarian-cancer or benign cases	General	3.1% (2)	64	Shaw et al., 2009 [101]
		0.8% (4)	522	Rabban et al., 2014 [142]
		1.1% (3)	277	Seidman et al., 2016 [140]
Fallopian tubes from endometrial serous carcinoma cases	General	22.7% (5)	22	Jarboe et al., 2009 [47]
		21.8% (12)	55	Stewart et al., 2010 [143]
		14.3% (4)	28	Tang et al., 2012 [106]
		7.9% (3)	38	Tolcher et al., 2015 [144]
Fallopian tubes from endometrial carcinoma or hyperplasia cases	General	1.7% (3)	175	Seidman et al., 2016 [140]

?: information not described in the cited reference.
